# Integrated Duo Wavelength VCSEL Using an Electrically Pumped GaInAs/AlGaAs 980 nm Cavity at the Bottom and an Optically Pumped GaInAs/AlGaInAs 1550 nm Cavity on the Top

**DOI:** 10.1155/2014/627165

**Published:** 2014-10-28

**Authors:** Samiha Ishrat Islam, Arnob Islam, Saiful Islam

**Affiliations:** ^1^Electrical and Electronic Engineering Department, American International University-Bangladesh, Dhaka 1213, Bangladesh; ^2^Electrical and Electronic Engineering Department, Northern University Bangladesh, Dhaka 1213, Bangladesh; ^3^Electrical and Electronic Engineering Department, Bangladesh University of Engineering and Technology, Dhaka 1000, Bangladesh

## Abstract

In this work, an integrated single chip dual cavity VCSEL has been designed which comprises an electrically pumped 980 nm bottom VCSEL section fabricated using GaInAs/AlGaAs MQW active region and a 1550 nm top VCSEL section constructed using GaInAs/AlGaInAs MQW active region but optically pumped using half of the produced 980 nm light entering into it from the electrically pumped bottom cavity. In this design, the active region of the intracavity structure 980 nm VCSEL consists of 3 quantum wells (QWs) using Ga_0.847_In_0.153_As, 2 barriers using Al_0.03_Ga_0.97_As, and 2 separate confinement heterostructures (SCH) using the same material as the barrier. The active region of the top emitting 1550 nm VCSEL consists of 3 QWs using Ga_0.47_In_0.52_As, 2 barriers using Al_0.3_Ga_0.17_In_0.53_As, and 2 SCHs using the same material as the barrier. The top DBR and the bottom DBR mirror systems of the 1550 nm VCSEL section plus the top and bottom DBR mirror systems of the 980 nm VCSEL section have been formed using GaAs/Al_0.8_Ga_0.2_As. Computations show that the VCSEL is capable of producing 8.5 mW of power at 980 nm from the bottom side and 2 mW of power at the 1550 nm from top side.

## 1. Introduction

It is well known that 850 nm–980 nm (short wavelength) and 1300 nm–1550 nm (long wavelength) MQW edge emitting diode lasers are widely used in optical communication systems [1, 2]. For these wavelengths, VCSELs are much better because of the many well-known advantages of VCSELs [3]. Conventional VCSELs are electrically pumped by injection current. In such a device, undesirable heating happens due to the flow of injection current through the layers of materials of the top and bottom DBR mirror systems. To eliminate this unwanted heating, intracavity structure has been used by the designers although this may introduce slight complexity in fabricating the VCSELs.

VCSEL with good beam quality (single transverse mode) greatly reduces the complexity of beam-shaping optics (compared to edge emitters) and increases the coupling efficiency to the fiber or pumped medium [4]. Unfortunately, poor beam quality is obtained from conventional electrically pumped VCSELs due to lasing of higher order transverse modes with even relatively small active diameters [4]. A transverse single-mode operation from large-area devices (large beam diameter) is always advantageous since such devices can provide low thermal resistance and high output power at small divergence angles [4].

In an electrically pumped VCSEL, it is not possible to inject carriers uniformly across a wide area for a large beam diameter in the laser cavity by traditional diode current injection [4]. As a result, other transverse modes are generated beside fundamental transverse mode which in turn degrades beam quality of the laser [4]. In order to mitigate this problem, a thick doped semiconductor current spreading layer can be used. But such a doped layer has strong free carrier absorption inside the extended laser cavity, which can degrade laser threshold and efficiency [4].

Using selective oxidation, single transverse mode in small aperture VCSEL may be obtained using very small diameter aperture with low injection current [5]. With high electrical pump current, beam quality significantly degrades [5]. So, practically it is almost impossible to have single transverse mode from large diameter VCSELs by electrical injection.

As an alternative to electrical pumping in VCSELs, optical pumping may be used which can inject excitation carriers uniformly across a wide area [4, 6]. In optical pumping, it is possible to obtain only fundamental transverse mode (TEM_00_) due to uniform injection of carriers. As a result, a very good beam quality can be obtained by using optical pumping. Moreover, self-heating is reduced by optical pumping to inject carriers directly into the active region, resulting in increased output power and lower operating temperature [6]. For optical pumping of a VCSEL, one requires a diode pump laser at a short wavelength for pumping a higher wavelength VCSEL. This system usually requires external setup for the pump laser and also for placing an external cavity mirror which requires external space as in OPSL/VECSEL/disk laser [7–9]. In such cases, efficient utilization of the pump light is also not simple.

Thus, the technique of optical pumping in lasers has been used for increasing beam power, beam brightness, and beam quality [8, 10–12]. Optically pumped VCSELs referred to as disk lasers or VECSELs (vertical external cavity surface emitting lasers), which are known to be power scalable laser sources, demonstrate very high beam quality and show similar performance as a diode pumped solid state laser (DPSS) but with lesser output power. Optically pumped VCSEL can operate over a very broad pump wavelength range [8]. Here, the pump-diode wavelength should be such that the band gap of the active region of the pump diode exceeds the band gap of the absorption layers of the optically pumped VCSEL.

In the design presented in this paper, optical pumping is achieved internally inside a VCSEL instead of externally, that is, in a different way than the OPSL/VECSEL/disk laser technique.

The proposed internal optically pumped VCSEL is expected to produce high beam quality and brightness by using the proposed optically pumped VCSEL although the output power may not be as high as an OPSL/VECSEL/disk laser. In this technique, the pump light is efficiently used because of the internal construction.

The motivation for this work arose after seeing the structures and ideas presented in the patents of Jayaraman [13] and Tan et al. [14]. However, detailed designs of the complete VCSELs are not available in the abovementioned publications. The design presented in this work is based on one of the ideas presented in the patent of Jayaraman [13].

In the theoretical design presented in this paper, a top emitting 1550 nm VCSEL which also emits 980 nm light from the bottom has been designed. The 1550 nm active absorber region of the integrated VCSEL is optically pumped through its bottom by an electrically pumped 980 nm VCSEL. During fabrication, the electrically pumped 980 nm VCSEL will be first fabricated on a GaAs substrate and then the bottom DBR of the 1550 nm VCSEL will be fabricated on top of it using epitaxial process. The cavity region of the 1550 nm VCSEL will be epitaxially grown separately on another GaAs substrate, which will next be etched off and the 1550 nm cavity region will then be wafer-bonded with the already grown 1550 nm bottom DBR mirror system. The 1550 nm top DBR mirror system together with a 980 nm additional top mirror system is to be grown separately using GaAs/Al_0.8_Ga_0.2_As and then wafer-bonded with the top side of the 1550 nm cavity system because of lattice mismatch. However, in the portions of the integrated VCSEL on top of the 1550 nm cavity and on the bottom of the cavity, the layers are almost lattice matched which makes the device attractive for epitaxial growth except the two wafer bonded regions. The two VCSEL sections are thus vertically integrated into a single device.

The design aims at mostly lattice matched epitaxial fabrication except two unavoidable wafer fused layers in the top and the bottom of the 1550 nm active absorber region.

Such a VCSEL will be useful in an optical communication system which uses both 980 nm and 1550 nm lights.

## 2. The Proposed Dual Cavity VCSEL Structure

The proposed VCSEL consists of a 1550 nm active cavity having top and bottom DBR mirror systems sitting on top of a 980 nm electrically pumped intracavity VCSEL structure made up of a 980 nm active MQW cavity with top and bottom GaAs/Al_0.8_Ga_0.2_As DBR mirror systems and grown on a GaAs substrate. On top of these two VCSEL sections another 980 nm GaAs/Al_0.8_Ga_0.2_As DBR mirror system is grown on the 1550 nm top mirror system.

The bottom 980 nm VCSEL is electrically pumped to produce 980 nm light output, a portion (50%) of which will come out from the bottom of the VCSEL. The remaining portion of the 980 nm light will come out of the top side, pass through the bottom GaAs/Al_0.8_Ga_0.2_As 1550 nm DBR mirror system, and enter into the 1550 nm MQW active region termed as the active absorber region. Here the 980 nm light will be absorbed as the 1550 nm active region has lower band gap. If adequate amount of light is available for absorption, the process will generate 1550 nm laser output because of the design and the top and bottom mirror systems.

The 1550 nm laser output, which is generated by the optical pumping of the 980 nm laser output from the bottom electrically pumped 980 nm laser, will come out from the top of the dual cavity integrated VCSEL. The 1550 nm light will not be able to come out from the bottom because of the 99.9% reflectivity of the 1550 nm bottom GaAs/Al_0.8_Ga_0.2_As DBR mirror system and the 980 nm laser. However, there is a chance that some 980 nm laser light may come out from the top along with the 1550 nm laser output if it is not fully absorbed. To take care of this situation, another 980 nm DBR mirror system is placed on top of the top 1550 nm DBR mirror system.

In the end, 1550 nm laser output will come out from the top of the VCSEL and 980 nm laser output will come out through the bottom of the dual cavity VCSEL. Both the wavelengths are widely used in communication systems.

## 3. Energy Subband and Gain Calculation

Following the conventional procedure, the discrete energy levels in the quantum well region have been computed using the well-known time independent Schrodinger equation under effective mass approximations and with the approximation of parabolic band nature [15]. Consider the following:

(1) $$\begin{array}{c} -\frac{\hbar^{2}}{2m_{\text{eff}}}\nabla^{2}\Psi +V\Psi =E\Psi , \end{array}$$

where Ψ is the particle wave function, *ħ* is the Planck's constant divided by 2*π*,  *m*_eff_ is the effective mass of the carrier, *V* is band potential, and *E* is energy levels of conduction or valence band.

The gain spectra of the MQW semiconductor lasers have been obtained through computations utilizing the well-known energy and density dependent broadening [16] gain model. Consider the following:

(2) g(E)=g0(E)|Mb|2 ×∑j,n∫Eg,jnEg,bρr,jn(E′)hhhhhhh×AijCij(fc−fv)E′,jnL(E−E′)dE′.

Here, *g*_0_ is gain prefactor [11], *M*_*b*_ is the average, energy independent, and momentum transition matrix element for the dipole transition in the bulk semiconductor [9], *ρ*_*r*,*jn*_ is the volume density states [11], *f*_*c*_ and *f*_*v*_ are the electron quasi-Fermi level functions in the conduction and valence band, respectively, *j* and *n* are for *C* subband and *V* subband numbers [16], and *L*(*E* − *E*′) is the lineshape broadening factor [16].

## 4. Design of the 980 nm MQW Cavity

### 4.1. Design of the Materials of the Layers of the 980 nm Cavity of the Integrated VCSEL

The 980 nm VCSEL section is grown on GaAs substrate. The energy gap, *E*_*g*_, of the quantum well material corresponding to the 980 nm laser is calculated to be 1.2685 eV. To achieve the abovementioned wavelength, the ternary compound semiconductor material GaInAs has been chosen. The composition of this ternary compound well material Ga_*x*_In_1−*x*_As to achieve a band gap of 1.19 eV is calculated using the equation given in Adachi [17]:

(3) $$\begin{array}{c} E_{g}=0.359+0.491x+0.580x^{2}. \end{array}$$

This gives the composition of Ga_0.847_In_0.153_As. To get a relatively higher band gap AlGaAs is chosen as the barrier material. To achieve an acceptable band offset the composition of the barrier material has been chosen to be Al_0.03_Ga_0.97_As. A band gap of 1.48 eV is calculated from this composition using the equation given in Adachi [17]:

(4) $$\begin{array}{c} E_{g}=1.43+1.707x-1.437x^{2}+1.310x^{3}. \end{array}$$

Here, the quantum well material is 1.1% compressively strained with respect to barrier material. Shift in conduction band bottom edge and the shift in the subbands (heavy hole and light hole) of valence band due to strain are computed using the analytical expressions available in [18]. Finally the above composition of well and barrier material produces a total transition energy of 1.2685 ev including conduction and valence band subbands.

The p- and n-cladding materials are chosen to be the same as Ga_0.51_In_0.49_P to obtain a band gap of 1.91 eV. The band gap of this composition is calculated using [17]

(5) $$\begin{array}{c} E_{g}=1.35+0.76x+0.65x^{2}. \end{array}$$

**Figure 1:**
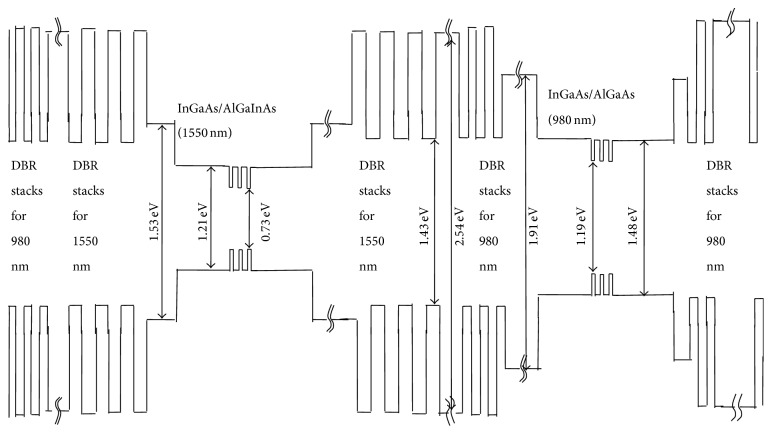
Energy band diagram showing the energy gaps at the different layers of the integrated optically pumped 1550 top emitting and electrically pumped 980 nm bottom emitting VCSELs. The substrate layer is on the right side (bottom) and the left side is the DBR layers of the 1550 nm VCSEL (top).

**Figure 2:**
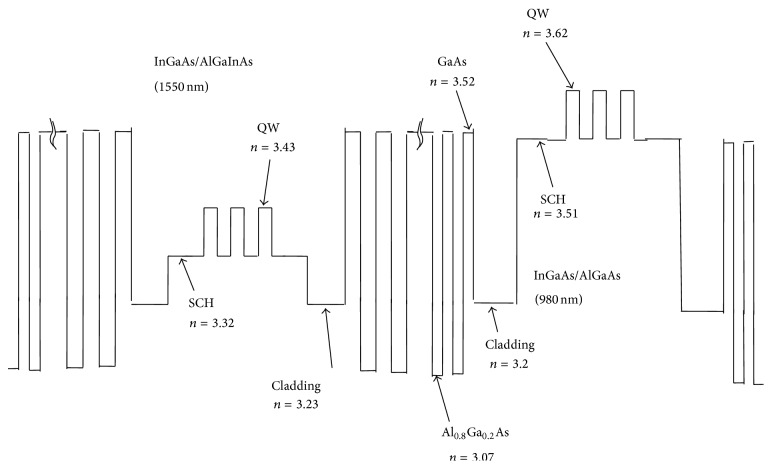
Values of the refractive indices of different layers of the integrated optically pumped 1550 top emitting and electrically pumped 980 nm bottom emitting VCSELs.

**Figure 3:**
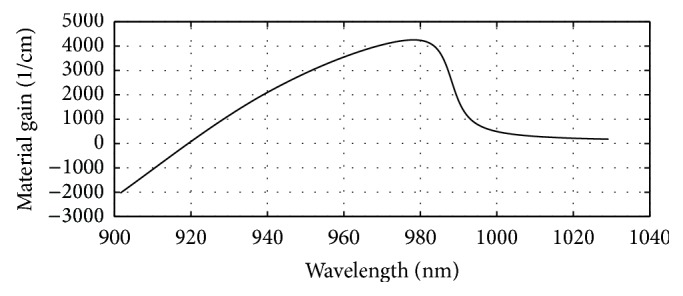
Plot of material gain versus wavelength for the designed 980 nm VCSEL cavity using Ga_0.847_In_0.153_As/Al_0.03_Ga_0.97_As active region showing peak gain at 980 nm.

The energy band profile of the layers of the 980 nm section VCSEL can be seen in Figure 1. The computed refractive indices of different layers of this section can be seen in Figure 2. The obtained plot of material gain versus wavelength of the 980 nm active region has been presented in Figure 3.

### 4.2. Computed Lattice Constants and Refractive Indices of the Compound Semiconductors Used in the 980 nm Cavity

Here, Ga_0.51_In_0.49_P has been used as both p- and n-cladding materials whose lattice constant is 5.653 Å and refractive index is 3.2. Al_0.03_Ga_0.97_As has been used as both SCH and barrier materials whose lattice constant is 5.666 Å and refractive index is 3.51. Ga_0.847_In_0.153_As has been used as the well material whose lattice constant is 5.7152 Å and refractive index is 3.62. The substrate is GaAs whose lattice constant is 5.653 Å and the refractive index is 3.52. The computed conduction band offset is 0.16 ev and the valence band offset is 0.129 ev. The lattice constant of the materials has been calculated using Vegard's law [19]:

(6) $$\begin{array}{c} a\left(A_{x}B_{1-x}C\right)=xa\left(AC\right)+\;\left(1-x\right)a\left(BC\right). \end{array}$$

For this 980 nm pump VCSEL section, intracavity structure has been chosen to reduce excess heat generation inside the cavity. Two oxide confinement layers have been incorporated for good confinement as shown in Figure 5.

## 5. Design of the 1550 nm MQW Cavity

### 5.1. Design of the Materials of the Layers of the 1550 nm Cavity of the Integrated VCSEL

The energy gap, *E*_*g*_, of the quantum well material corresponding to the 1550 nm laser is calculated to be 0.8019 eV. To achieve the abovementioned wavelength, the ternary compound semiconductor material GaInAs has been chosen. The composition of this ternary compound well material Ga_*x*_In_1−*x*_As to achieve a band gap of 0.729 eV is calculated using (3) [17]. This gives the composition of Ga_0.47_In_0.53_As which will produce a total band gap of 0.8019 including conduction and valence band subbands.

To achieve an acceptable band offset the barrier material has been chosen to be a quaternary compound Al_*x*_Ga_*y*_In_1−*x*−*y*_As (lattice matched to InP). The composition of the semiconductor Al_0.3_Ga_0.17_In_0.53_As is calculated using the equation given in Selmic et al. [20]. Consider the following:

(7) $$\begin{array}{c} E_{g}=0.75+1.548x,\\ 1-x-y=0.53. \end{array}$$

This barrier material produces a band gap of 1.48 eV.

In this case the lattice constant of the quantum well material is almost matched with the lattice constant of 5.8690 Å of the barrier material. The number of quantum wells chosen for this design is 3. The p-cladding material (*E*_*g*_ = 1.53 eV) is chosen as Al_0.48_In_0.52_As (Si doped) and the n-cladding material is chosen as Al_0.48_In_0.52_As (C doped) (*E*_*g*_ = 1.53 eV); both are lattice matched. The energy gap for this composition of the materials has been computed using [17]

(8) $$\begin{array}{c} E_{g}=0.359+1.931x+0.72x^{2}. \end{array}$$

**Figure 4:**
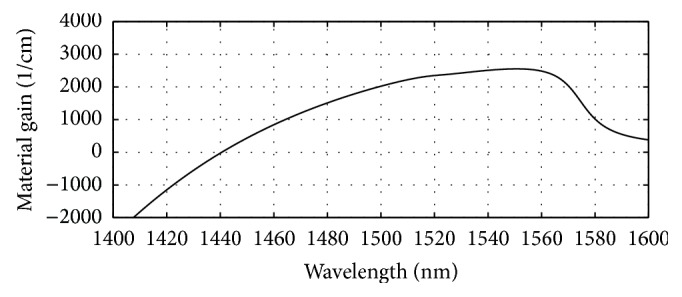
Plot of material gain versus wavelength for the designed 1550 nm VCSEL cavity using Ga_0.47_In_0.53_As/Al_0.3_Ga_0.17_In_0.53_As active region showing peak gain at 1550 nm.

**Figure 5:**
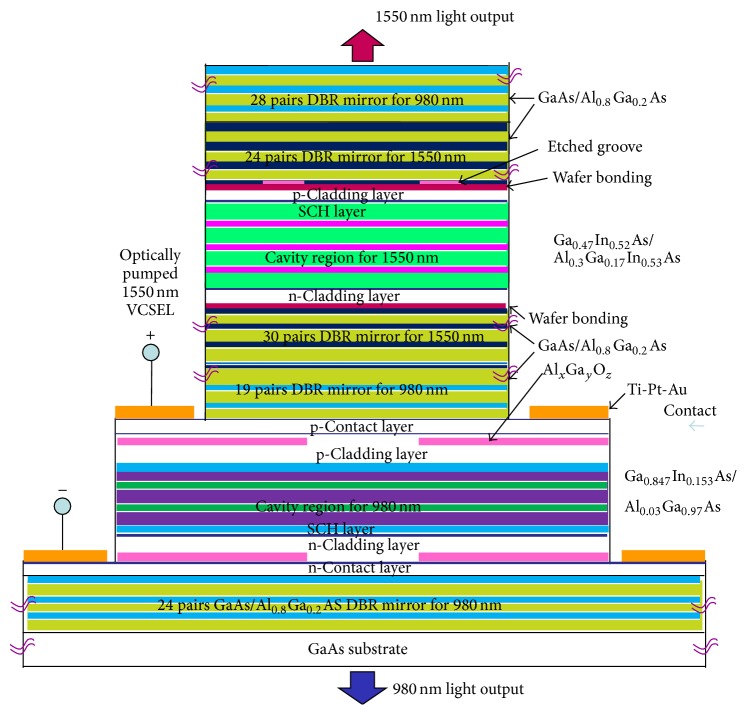
The structure of the designed top emitting 1550 nm VCSEL optically pumped using a 980 nm intracavity VCSEL, showing the materials used in the different layers. The 980 nm VCSEL is to be fabricated on a GaAs substrate and the 1550 nm VCSEL is to be grown on top of the 980 nm VCSEL.

The energy band profile of the layers of the 1550 nm section VCSEL can be seen in Figure 1. The computed refractive indices of different layers of this section can be seen in Figure 2. The obtained plot of material gain versus wavelength for the 1550 nm active region has been presented in Figure 4. The designed integrated VCSEL structure is shown in Figure 5.

### 5.2. Computed Lattice Constants and Refractive Indices of the Compound Semiconductors Used in the 1550 nm Cavity

For both the p- and n-cladding layers Al_0.48_In_0.52_As has been used whose lattice constant is 5.8690 Å and refractive index is 3.23. For both the SCH and barrier layers, Al_0.3_Ga_0.17_In_0.53_As has been used whose lattice constant is 5.8690 Å and refractive index is 3.32. Ga_0.47_In_0.53_As has been used as the well material whose lattice constant is 5.8690 Å and refractive index is 3.43. This 1550 nm active portion has to be grown on an InP substrate whose lattice constant is 5.8690 Å and refractive index is 3.18. The computed conduction band offset of this well/barrier combination is 0.3575 ev and the valence band offset is 0.139 ev. The lattice constant of the materials has been calculated using Vegard's law [19] presented in (6).

## 6. Design of the DBR Mirror Systems 

### 6.1. Design of the 980 nm DBR Mirror System

**Figure 6:**
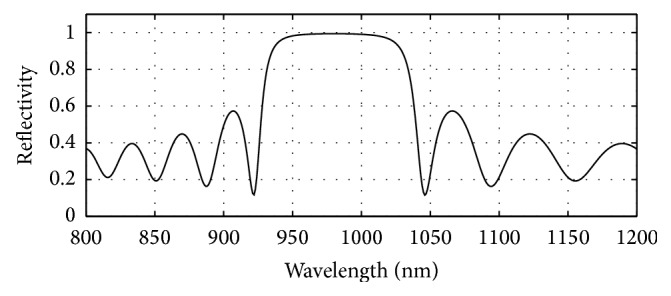
Plot of reflectivity versus wavelength for the top DBR mirror system of the designed bottom 980 nm VCSEL. 19 pairs of GaAs/Al_0.8 _Ga_0.2 _As gave 0.994 reflectivity.

**Figure 7:**
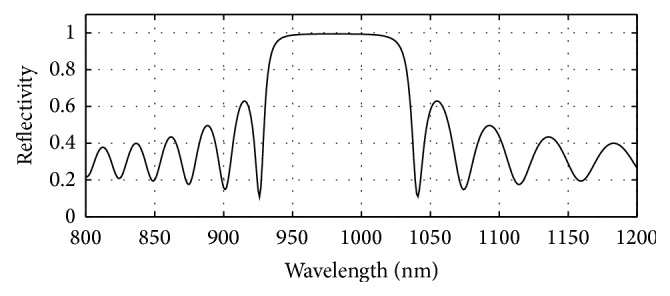
Plot of reflectivity versus wavelength for the bottom DBR mirror system of the designed bottom 980 nm VCSEL. Here, 24 pairs of GaAs/Al_0.8 _Ga_0.2 _As gave 0.994 reflectivity.

**Figure 8:**
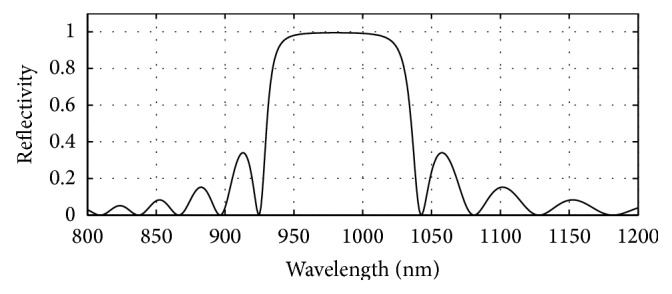
Plot of reflectivity versus wavelength for the 980 nm DBR mirror system placed on top of the designed top 1550 nm VCSEL section. 28 pairs of GaAs/Al_0.8_Ga_0.2_As gave 0.994 reflectivity.

GaAs (3.52)/Al_0.8 _Ga_0.2 _As (3.07) has been used in the bottom as well as in the top mirror systems of the designed bottom 980 nm VCSEL section. The plots of reflectivity versus wavelength obtained after computation of the reflectivities of the mirror systems using the analytical expressions given in [21] are presented in Figures 6, 7, and 8. The top mirror system of the 980 nm VCSEL section has been designed to achieve a reflectivity of 99.4% (Figure 6) and the bottom mirror system for 99.4% (Figure 7) for getting equal amount of light emission from top and bottom sides.

A mirror system has been placed at the top of the top most 1550 nm DBR mirror system whose reflectivity is set to 99.4%. The values of thickness of the quarter wave GaAs (3.52)/Al_0.8_Ga_0.2_As (3.07) layers are computed as 69.6 nm/79.8 nm.

### 6.2. Design of the 1550 nm DBR Mirror System

**Figure 9:**
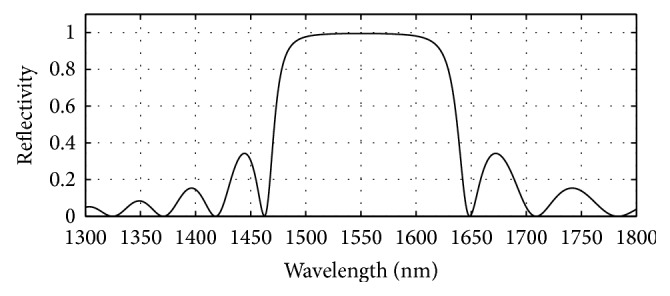
Plot of reflectivity versus wavelength for the bottom DBR mirror system of the designed top 1550 nm VCSEL section. 30 pairs of GaAs/Al_0.8_Ga_0.2_As produced 0.999 reflectivity.

**Figure 10:**
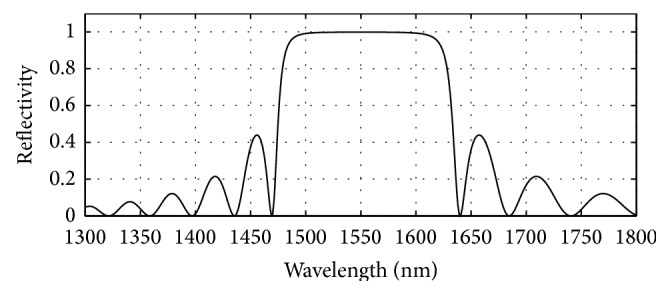
Plot of reflectivity versus wavelength for the top DBR mirror system of the designed top 1550 nm VCSEL section. 24 pairs of GaAs/Al_0.8_Ga_0.2_As gave 0.994 reflectivity.

The materials chosen for the top as well as the bottom mirror systems GaAs (3.52)/Al_0.8_Ga_0.2_As (3.07) of the 1550 nm VCSEL section are the same as the DBR mirror systems of the 980 nm VCSEL section. The values of thickness of the quarter wave GaAs (3.52)/Al_0.8_Ga_0.2_As (3.07) layers are computed as 126.2 nm/110.1 nm. The top mirror system has been designed to achieve a reflectivity of 99.4% and the bottom mirror system for 99.9%. The plots obtained after computation of the reflectivities are presented in Figures 9 and 10.

An etched groove has been created in the bottom of the top DBR mirror system to obtain air post action [13] for confinement instead of using oxide confinement (shown in Figure 5).

## 7. Design of the Thickness of the Layers of the Integrated VCSEL

### 7.1. Thickness Values of the Different Layers of the 980 nm Section of the Integrated VCSEL

The cavity length has been chosen to be 1.5 *λ* with the three quantum wells centered at the antinode of the laser optical field standing wave and the cavity radius has been taken as 5 *μ*m. The thickness of the well (Ga_0.847_In_0.153_As) has been chosen as 7 nm which corresponds to an optical thickness of 25.34 nm. The thickness of the barrier (Al_0.03_Ga_0.97_As) has been chosen as 8 nm which corresponds to an optical thickness of 28.08 nm. The thickness of the SCH (Al_0.03_Ga_0.97_As) has been chosen as 70 nm which corresponds to an optical thickness of 351 nm. The thickness of the p- as well as the n-cladding (Ga_0.51_In_0.49_P) has been chosen as 132 nm which corresponds to an optical thickness of 640 nm. The structure is symmetric. The cavity length is then obtained by adding the thicknesses of the layers as =1470 nm which is almost 1.5 wavelength.

### 7.2. Thickness Values of the Different Layers of the 1550 nm Section of the Integrated VCSEL

In this section also the cavity length has been chosen to be 1.5 *λ* with three quantum wells and the cavity radius has been taken as 10 *μ*m. The thickness of the well (Ga_0.47_In_0.53_As) has been chosen as 7 nm which corresponds to an optical thickness of 24.01 nm. The thickness of the barrier (Al_0.3_Ga_0.17_In_0.53_As) has been chosen as 8 nm which corresponds to an optical thickness of 26.56 nm. The thickness of the SCH (Al_0.3_Ga_0.17_In_0.53_As) has been chosen as 122 nm which corresponds to an optical thickness of 405 nm. The thickness of the p- as well as the n-cladding (Al_0.48_In_0.52_As) has been chosen as 215 nm which corresponds to an optical thickness of 694 nm. The structure is symmetric. The cavity length is then obtained by adding the thicknesses of the layers as =2323 nm which is almost 1.5 wavelength (2325 nm).

## 8. Analytical Expressions for Computing Output Power of the Optically Pumped VCSEL

For the optically pumped 1550 nm VCSEL section the analytical expression of threshold pump power *P*_th_ presented in [11] can be used here as

(9) $$\begin{array}{c} P_{\text{th}}=N_{\text{th}}\frac{h\nu N_{w}L_{w}A_{p}}{\eta_{\text{abs}}\tau \left(N_{\text{th}}\right)}, \end{array}$$

where *L*_*w*_ = well thickness, *N*_*w*_ = number of wells, *A*_*p*_ = pump spot area, *η*_abs_ = pump absorption efficiency, and *τ*(*N*_th_) = carrier lifetime at threshold carrier density.

The threshold carrier density *N*_th_ in this case is [11]

(10) $$\begin{array}{c} N_{\text{th}}=N_{\mathrm{tr}}{\left(\frac{1}{R_{1}R_{2}T_{\text{loss}}}\right)}^{{\left(2\Gamma g_{0}N_{w}L_{w}\right)}^{-1}}, \end{array}$$

where *T*_loss_ = transmission factor due to round-trip cavity loss of optical wave inside the cavity and *R*_1_ and *R*_2_ are reflectivities of the bottom and top DBR mirror systems of the 1550 nm VCSEL section.

The output power of the pumped 1550 nm VCSEL section *P*_lase_ can be expressed as [11]

(11) $$\begin{array}{c} P_{\text{lase}}=\left(P_{p}-P_{\text{th}}\right)\eta_{\text{diff}}, \end{array}$$

where

(12) $$\begin{array}{c} \eta_{\text{diff}}=\eta_{\text{out}}\eta_{\text{quant}}\eta_{\text{abs}}. \end{array}$$

In this expression, the output efficiency is

(13) $$\begin{array}{c} \eta_{\text{out}}=\frac{\ln\left(R_{2}\right)}{\ln\left(R_{1}R_{2}T_{\text{loss}}\right)}. \end{array}$$

The quantum defect efficiency *η*_quant_ may be expressed as [11]

(14) $$\begin{array}{c} \eta_{\text{quant}}=\frac{\lambda_{\text{pump}}}{\lambda_{\text{laser}}}. \end{array}$$

Using these equations, the computation of optically pumped output power of the 1550 nm VCSEL section has been performed using the values of *η*_abs_ = 0.8 and *T*_loss_ = 0.999. The values of the reflectivities are taken from the design as *R*_1_ = 0.999 and *R*_2_ = 0.994.

## 9. Performance Characteristics of the Integrated VCSEL

After designing the desired integrated 980 nm pumped 1550 nm VCSEL, the performance parameters of this integrated VCSEL are computed through simulation. For this purpose at first, the solutions of the coupled rate equations for 980 nm electrically pumped laser have been obtained for a time window of 0–2.5 ns.

The coupled rate equations, (i) the rate equation for carrier density and (ii) the rate equation for photon density, are solved simultaneously for a chosen value of injection current of 6 mA (which is taken above the threshold current) using MATLAB.

**Figure 11:**
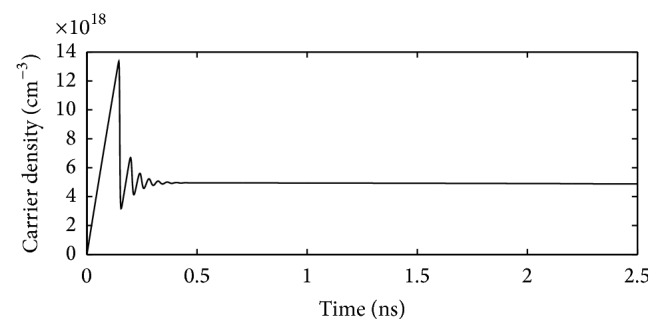
Plot of carrier density versus time for the designed 980 nm electrically pumped VCSEL.

Using the output of this computation work a plot of carrier density versus time is obtained for the designed 980 nm optically pumped VCSEL. This plot is presented in Figure 11.

**Figure 12:**
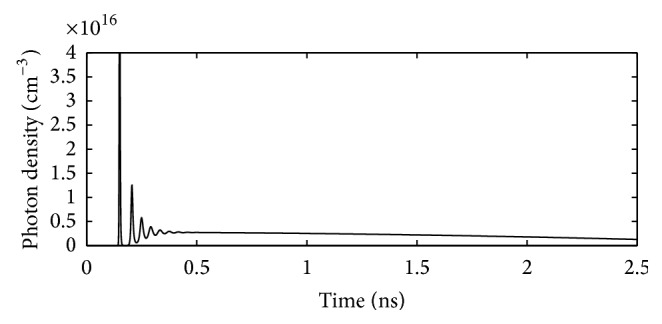
Plot of photon density versus time for the designed 980 nm electrically pumped VCSEL.

Using the output of the same computation work mentioned above, a plot of photon density versus time is obtained for the designed 980 nm VCSEL. This plot is presented in Figure 12.

The output power of the 980 nm pump laser is calculated using the following equation:

(15) $$\begin{array}{c} P_{\text{out}}=\frac{v_{g}\alpha_{m}h\upsilon V_{a}S}{\Gamma }\left(1-\frac{\Delta T}{\Delta T_{\mathrm{off}}}\right), \end{array}$$

where *v*_*g*_ = group velocity, *α*_*m*_ = mirror loss, *V*_*a*_ = active region volume, *S* = photon density, and Γ = confinement factor. Here (1 − Δ*T*/Δ*T*_*off*⁡_) is the empirical injection efficiency factor [22] which takes into account the temperature dependent effects or thermal rollover, where Δ*T* = internal temperature rise and Δ*T*_*off*⁡_ = turn-off temperature rise.

**Figure 13:**
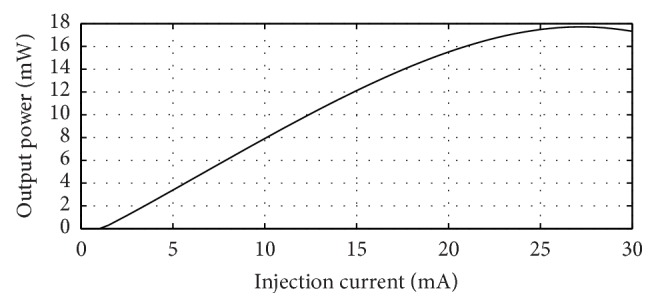
Plot of output power versus injection current for the designed 980 nm electrically pumped VCSEL showing a threshold current of 1.5 mA at room temperature (27°C). For an injection current value of 25 mA in the 980 nm VCSEL section 17 mw power is produced of which 8.5 mw will go to the top side which will cause optical pumping and the rest 8.5 mw of the 980 nm power will come out from the bottom.

Using this equation and the parameter values presented above a plot of the 980 nm output power versus injection current of the designed 980 nm VCSEL is obtained through computation. This plot is presented in Figure 13.

**Figure 14:**
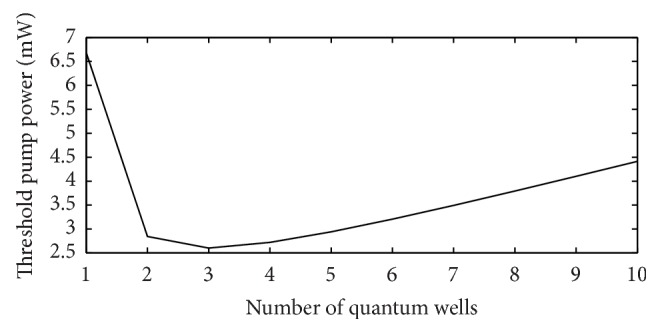
Plot of threshold pump power versus the number of quantum wells for the designed 1550 nm electrically pumped VCSEL showing threshold pump power minimum using three quantum wells.

The choice of the number of quantum wells in 1550 nm VCSEL section is critical as it affects the threshold pump power. The pump power which is obtained from the 980 nm VCSEL section of this design is limited to about 8.5 mw. As it is not possible to increase the pump power, the only way to get maximum possible output power is to minimize the threshold pump power as much as possible. It has been found that there is an optimum number of quantum wells for which threshold pump power is minimum.  Figure 14 shows that the minimum threshold power is obtained when there are three quantum wells in the optically pumped 1550 nm section.

**Figure 15:**
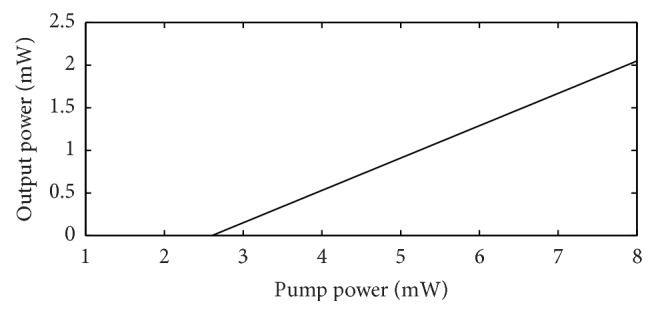
Plot of output optical power (1550 nm) versus pump power (980 nm) for the designed optically pumped 1550 nm VCSEL.

The output power of the 1550 nm VCSEL section optically pumped by the bottom 980 nm VCSEL section is calculated using (9)–(14) presented above. Using these equations and the parameter values presented above a plot of output power versus input 980 nm pump light power for the designed 1550 nm VCSEL is obtained through computation. This plot is presented in Figure 15.

In the presented design, 17 mW of output power of the 980 nm pump VCSEL can be obtained at 25 mA (Figure 13) injection current. 8.5 mw of this power will come out from the bottom and 8.5 mw will come out from the top and will be available for pumping the 1550 nm absorber active region because of the same amount of reflectivity of the top and bottom 980 nm DBRs. From the plot of the 1550 nm output power versus 980 nm pump power of Figure 14, it is seen that a pump power of about 8 mw produces about 2 mw of 1550 nm optically pumped output power.

## 10. Conclusions 

In this work, well-known and widely used GaAs/Al_0.8_Ga_0.2_As materials have been used in the top and bottom DBR mirror systems for both the 1550 nm and the 980 nm VCSEL sections except the obvious change in thickness. The well and barrier regions of the 980 nm VCSEL section have small lattice mismatch which has been ignored. The only section where lattice matching could not be obtained is the 1550 nm active region (Ga_0.47_In_0.53_As/Al_0.3_Ga_0.17_In_0.53_As) with its DBR mirror system (GaAs/Al_0.8_Ga_0.2_As) due to which wafer fusion has to be applied on the top and bottom sides of this active region. The 1550 nm top DBR mirror system and the 980 nm topmost DBR mirror system are to be grown separately on a GaAs substrate using epitaxial process. A circular etched groove is formed at the bottom of the 1550 nm mirror system for optical confinement instead of forming oxide layer. The Ga_0.47_In_0.53_As/Al_0.3_Ga_0.17_In_0.53_As 1550 nm active region is grown separately on an InP substrate and the substrate is then etched off. The 1550 nm active region is then wafer-fused on the top and bottom sides to obtain the integrated optically pumped VCSEL.

The performance characteristic curves of both of the sections show acceptable performance. For this design, computations show that the electrically pumped 980 nm VCSEL section has a threshold current of 1.5 mA and is capable of giving 17 mw at a current of 25 mA. Design has been made to get 8.5 mw of this power from the bottom of the 980 nm VCSEL section and the rest 8.5 mw is available for optically pumping the top positioned 1550 nm VCSEL section which then produces an output power of 2 mW. The important thing to be noted here is that the pump power in this structure is delivered precisely where it is needed without any loss. It is possible to obtain high beam quality and high brightness beam using such an integrated optical pumped VCSEL but unfortunately with the present design increasing the power significantly appears to be a difficult job due to difficulty in heat extraction from the cavity. The presented design of the integrated VCSEL is based upon simple structure and widely used materials in the active as well as in the DBR layers. The integrated VCSEL is expected to perform well after fabrication.

Except the patents of Jayaraman [13] and Tan et al. [14] mentioned in Section 1, no research paper could be found, using common search techniques, which present performance results from a fabricated duo wavelength producing VCSEL. No other published paper could be found which contains theoretical analysis based performance characteristics of a duo wavelength producing VCSEL.
